# A 16-year-old female with Kala Pathar poisoning, complicated by uncommon cardiac and hepatic manifestations: a rare case report of intensive care management

**DOI:** 10.1097/MS9.0000000000005262

**Published:** 2026-06-15

**Authors:** Muhammad Asif, Fnu Muhammad, Muhammad Ilyas, Sawera Tahir, Iqra Sarwar, Muddassir Khalid

**Affiliations:** a Department of Medicine, Benazir Bhutto Hospital, Rawalpindi, Pakistan; b Department of Medicine, Rawalpindi Medical University, Rawalpindi, Pakistan; c Department of Medicine, Daisy Hill Hospital, Northern Ireland, Ireland; d Department of Medicine, Ysbyty Gwynedd Bangor, Wales, United Kingdom; e Department of Biochemistry and Molecular Biology, NUMS Army Medical College, Rawalpindi, Pakistan; f Department of Medicine, Nishtar Medical University, Multan, Pakistan

**Keywords:** angioedema, hair dye, Kala Pathar, paraphenylenediamine, poisoning, PPD, pseudomediastinum

## Abstract

**Introduction and importance:**

In the subcontinent, hair color containing paraphenylenediamine (PPD) is frequently used due to its affordability and easy accessibility. When administered topically or consumed, PPD has harmful effects both locally and systemically. Since there is no known cure, early detection and supportive interventions are the cornerstones of care.

**Case presentation:**

We now describe the effective treatment of a young female patient who experienced angioedema, cardiac manifestations, and hepatic dysfunction following PPD ingestion. Clinical signs such as angioedema and dark brown urine may indicate PPD toxicity in the absence of testing facilities.

**Clinical discussion:**

When taken orally, it is highly hazardous, and the results are primarily dependent on the dosage. Angioedema resulting in dysphagia and respiratory distress, hepatic necrosis, rhabdomyolysis, intravascular hemolysis, and abrupt renal failure are significant clinical symptoms. In PPD poisoning, myocarditis or deadly arrhythmias are also possible outcomes.

**Conclusion:**

Healthcare providers should maintain vigilance in regions where PPD exposure is prevalent, and public awareness and product regulation measures are necessary to mitigate the risks associated with PPD-containing products.

## Introduction

Paraphenylenediamine (PPD), a derivative of p-nitroaniline, is a standard oxidizable hair color. In Africa, the Middle East, and the Indian subcontinent, it is frequently used in conjunction with henna, which is traditionally applied to color the palms of the hands, soles of the feet, and hair a deep shade of red^[^1,2^]^. When administered topically or consumed, PPD has harmful effects both locally and systemically. When taken orally, it is highly hazardous, and the results are primarily dependent on the dosage – estimates of PPD’s fatal dosage range from 7 to 10 g^[^3^]^. A significant dosage will result in death from either cardiotoxicity (fatal arrhythmias) or angioneurotic edema within the first 6–24 hours of intake^[^4,5^]^. In most cases, angioneurotic edema is the only side effect that appears after smaller dosages or if the patient vomits the majority of the dye. Within the first week, a modest dosage will result in acute renal failure.

HIGHLIGHTS

- Paraphenylenediamine (PPD) (Kala Pathar) ingestion is an emerging toxicological emergency in South Asia.
- Clinical features include angioedema, hepatic injury, and cardiac involvement.
- Our patient developed rare hepatic and myocardial complications after ingestion.
- Early intensive care unit care, airway management, and supportive therapy ensured survival.
- Vigilance, regulation, and awareness are vital to reducing PPD-related morbidity.

Although upper airway edema and renal dysfunction are widely recognized in PPD intoxication, concurrent cardiac biomarker elevation, severe hepatocellular injury, and pseudomediastinal thoracic complications are rarely documented together. A focused literature review was also undertaken to compare this presentation with previously reported uncommon complications of PPD toxicity. We report this case to highlight the rare simultaneous occurrence of cardiac enzyme elevation, severe hepatic dysfunction, and pseudomediastinal complications in PPD poisoning, while also emphasizing the critical role of prompt airway protection and intensive supportive care. Such multisystem involvement in a single patient is rarely documented and carries important implications for prolonged monitoring and critical care management beyond initial airway stabilization.

## Case presentation

A 16-year-old female presented to us after ingesting an unknown quantity of PPD 3 hours ago, followed by multiple episodes of vomiting, tonic-clonic seizures, and frothing. On presentation in the emergency department, she exhibited swelling of the face, lips, and neck, along with dysphagia, suggestive of angioedema within 3 hours of ingestion. Upon admission, she had a respiratory rate of 18 breaths/min, a blood pressure of 130/80 mmHg, and a pulse rate of 100 beats per minute, with an SpO_2_ of 98% and a random blood sugar level of 212 mg/dl. On examination, mild, bilateral, scattered crepitations were heard in the lung fields. Other general and systemic clinical examinations were within normal limits.

### Investigations

Routine investigations, including blood complete picture, serum electrolytes, liver function tests, renal function tests, clotting profile, and specific investigations, including serum calcium, serum magnesium, CPK levels, albumin, troponin I, abdominal sonography, and total proteins, were performed. Hemogram, urine examination, and kidney function tests, along with serum electrolytes, were within normal limits at the time of admission and during the hospital stay. Serial liver function tests revealed progressive elevation of aspartate aminotransferase levels up to a maximum of 1363 on the 5th day and alanine aminotransferase levels up to a maximum of 4490 U/L on the 2nd day, with normal bilirubin. Her ultrasound of the abdomen and pelvis showed liver congestion, while the spleen and kidneys were normal. Right pleural effusion was noted. Other investigations were as follows: serum calcium: 8.4 mg/dl, serum magnesium: 2.2 mg/dl, troponin I: 1.19 ng/dl, CPK: 925.55, pleural fluid R/E: turbid, increased RBC (hemomediastinum), pleural fluid culture and sensitivity showed no growth obtained in the medical emergency room.

### Management

Emergency room management included providing information to the police for a medicolegal case. Gastric lavage was performed using activated charcoal. Injectable medications, including omeprazole, calcium gluconate, sodium bicarbonate, and hydrocortisone, were administered. An infusion of normal saline was also given. An ECG was performed, which showed no abnormalities. A call for consultation was sent to the intensive care unit (ICU) and the Otorhinolaryngology department. An elective tracheostomy was performed due to the expected laryngeal edema. The patient was then transferred to the ward, where they were received in the ICU. In the ICU, medications included omeprazole, heparin, vancomycin, tigecycline, tazobactam, potassium replacement, calcium gluconate, nebulization with ipratropium and beclomethasone, and limb and chest physiotherapy. The patient remained on ventilatory support and was shifted to CPAP on the 10th day, after which she showed gradual improvement. Broad-spectrum antibiotics were initiated empirically because of prolonged ventilatory support, chest tube placement, and concern for secondary pulmonary infection in the setting of pleural collection, despite subsequent sterile pleural cultures. Prophylactic low-dose heparin was administered because of immobilization and extended intensive care stay to reduce venous thromboembolic risk. The prolonged ICU admission was primarily necessitated by continued airway monitoring following tracheostomy, serial reassessment of pleural and mediastinal complications, and close observation of progressive hepatic and cardiac biochemical abnormalities.

### Outcomes and follow-up

**Figure 1.**
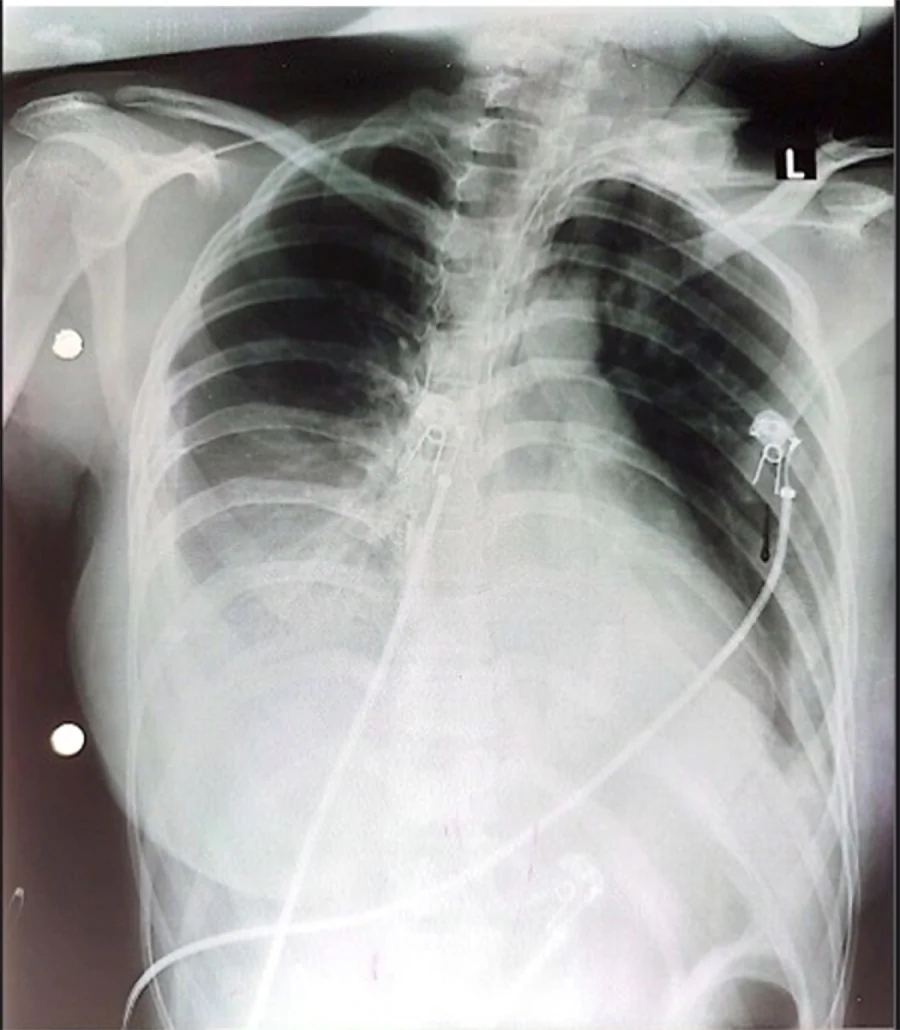
Chest radiograph showing right-sided pleural effusion.

**Figure 2.**
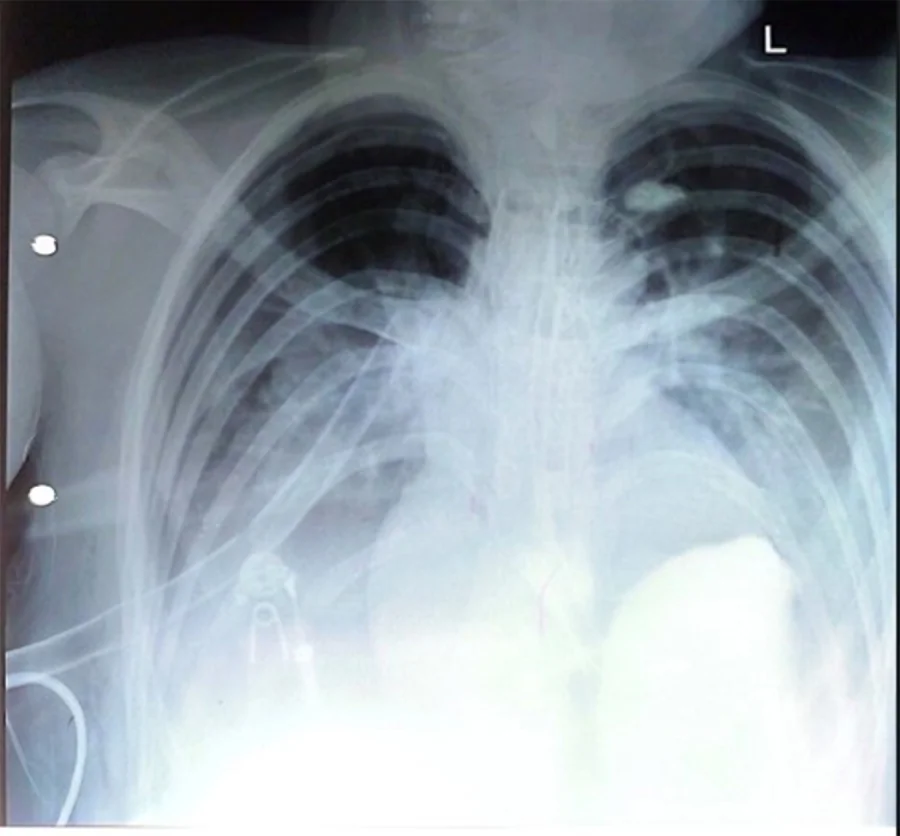
Post-chest tube insertion chest radiographs.

**Figure 3.**
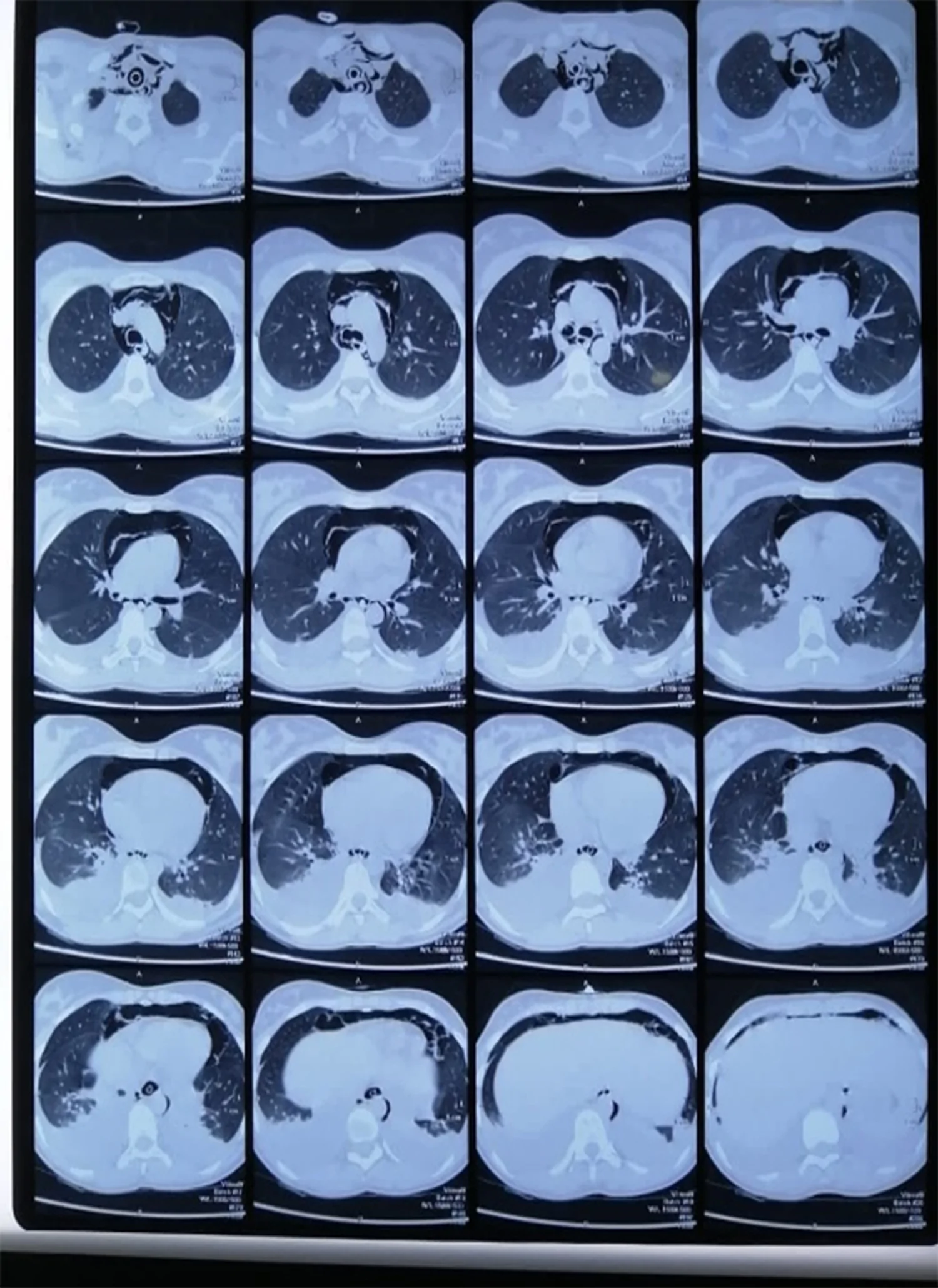
HRCT showing pneumomediastinum.

**Figure 4.**
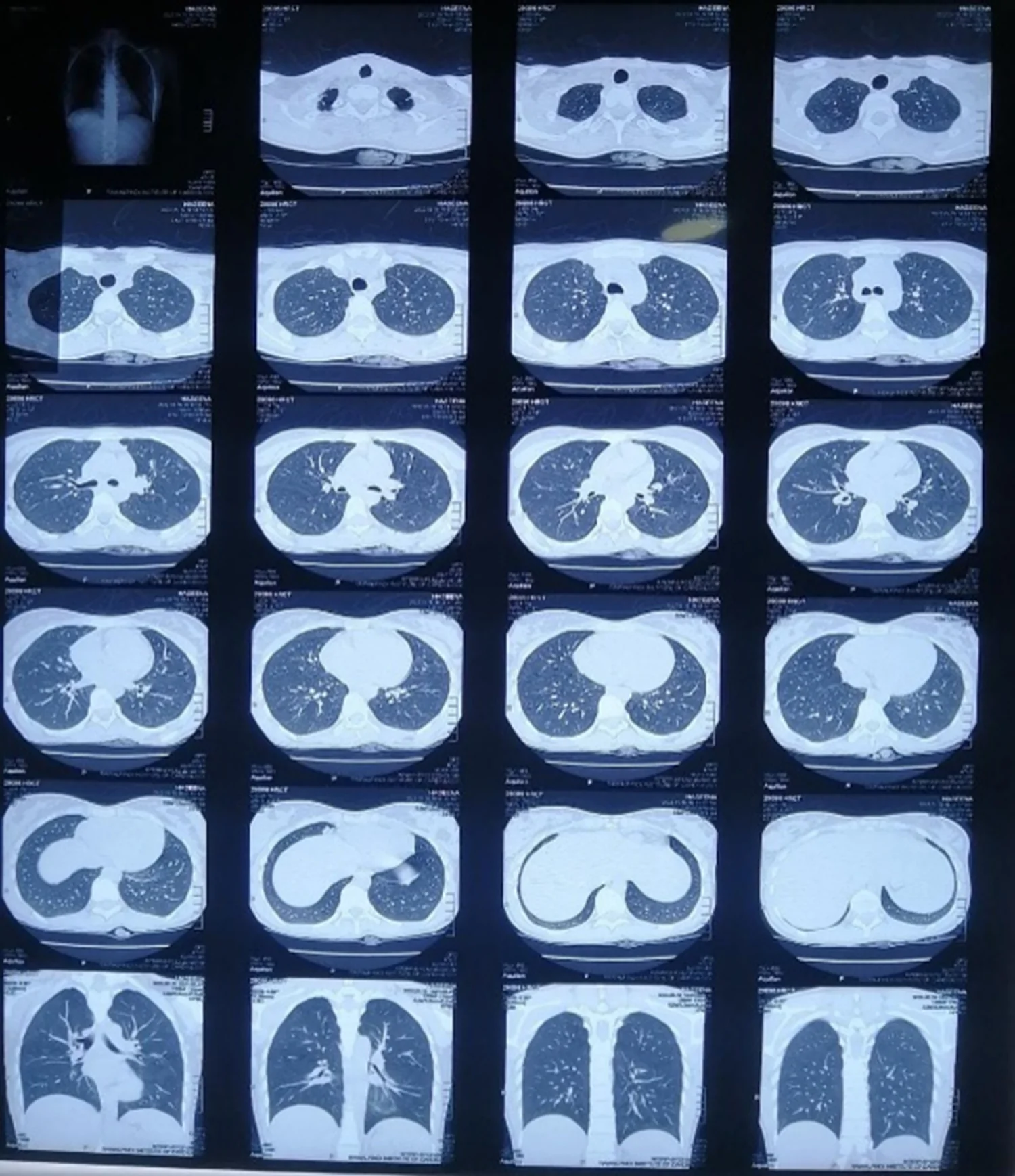
HRCT shows the resolution of pneumomediastinum.

On day 2, LFT derangement was noticed and managed accordingly, on day 3, pleural effusion right-sided was noted in chest radiograph shown in Figure 1 and a chest tube was inserted (post-chest tube insertion chest radiographs are shown in Figure 2, on day 4, echocardiography revealed possible pneumomediastinum/hemothorax for which HRCT chest was done (Fig. 3). For the pneumomediastinum, a surgical consult suggested ruling out an esophageal tear, for which contrast studies were carried out. An upper GI endoscopy was also performed; consequently, no varices, ulcers, or tears were observed. The study was unremarkable. From day 6 onward, the patient showed improvement in their condition. CPAP and T-piece trials were effectively tolerated. The patient remained in the ICU until the pleural effusion and pneumomediastinum resolved (Fig. 4), and the massively deranged LFTs normalized. The patient was then transferred to the medical ward, from which she was discharged. A summary timeline of major clinical events during hospitalization is shown in Table 1

**Table 1:** Summary timeline of major clinical events during hospitalization.

Hospital day	Major clinical events
Day 0	PPD ingestion, vomiting, seizures, angioedema, emergency tracheostomy, ICU admission
Day 2	Marked LFT derangement detected
Day 3	Right pleural effusion identified, chest tube inserted
Day 4	HRCT revealed pneumomediastinum/hemomediastinum, GI tear ruled out
Day 6 onward	Gradual respiratory and biochemical improvement
Day 10	Shifted to CPAP/T-piece trials
Subsequent days	Resolution of pleural/mediastinal findings and normalization of liver enzymes
Discharge	Stable discharge to ward then home

‘This case report has been prepared in line with the SCARE checklist^[^6^]^. In addition, manuscript preparation and literature synthesis were undertaken with adherence to contemporary reporting transparency recommendations in the era of artificial intelligence-assisted scientific writing^[^7^]^.

## Discussion

PPD poisoning is frequently reported from South Asia, the Middle East, and parts of Africa due to the widespread availability of hair dye preparations marketed as “Kala Pathar.” Although cervicofacial angioedema, rhabdomyolysis, and acute kidney injury are classical manifestations, the simultaneous presence of marked hepatocellular injury, biochemical myocardial involvement, pleural/mediastinal complications, and prolonged intensive care dependence remains distinctly uncommon in published reports. Previously documented cases have usually emphasized one dominant atypical complication – such as isolated myocarditis, fulminant hepatitis, or severe airway edema – whereas our patient demonstrated multiple uncommon systemic toxicities during a single admission, thereby making this presentation clinically noteworthy. Early signs of oral PPD consumption include burning and numbness in the mouth and throat, vomiting, upper GI tract swelling (which results in dysphagia), and respiratory distress brought on by angioedema and upper airway swelling^[^8,9^]^. These symptoms often appear 4–6 hours after ingestion. After 12 hours of consumption, a late phase typically begins if the patient survives the acute phase. It might linger for a few days or even a few weeks. Acute tubular necrosis/acute renal failure, oliguria/anuria, rhabdomyolysis, and intravascular hemolysis are delayed consequences in some individuals who survive the late phase^[^8^]^. The primary cause of renal failure, morbidity, and death is rhabdomyolysis^[^10^]^. Despite having consumed a fatal amount, our patient was fortunate to avoid developing renal failure, most likely as a result of prompt identification of the toxin and appropriate conservative care. Cardiac involvement in PPD poisoning has been infrequently described and may range from transient arrhythmias to toxic myocarditis and myocardial necrosis^[^11–14^]^. In our patient, the elevated troponin I level suggested myocardial injury; however, electrocardiography did not reveal acute ischemic changes^[^10,13^]^. Alternative causes of troponin elevation, including demand ischemia secondary to systemic stress, seizure-related catecholamine surge, or transient hypoxic myocardial strain, were also considered. Nevertheless, the absence of prior cardiac disease, the temporal association with acute poisoning, and spontaneous normalization of cardiac biomarkers favored toxin-related reversible myocardial involvement rather than primary coronary pathology. We have therefore revised the manuscript to describe this finding as probable toxic myocardial injury instead of definitive myocarditis^[^11,14^]^.

The development of pneumomediastinal and hemomediastinal changes in this patient was likely multifactorial, potentially related to forceful, repeated vomiting; seizure-associated abrupt intrathoracic pressure fluctuations; traumatic airway manipulation; or alveolar rupture in the setting of severe soft tissue edema. The rarity of this finding in PPD poisoning further complicated early diagnostic interpretation and necessitated the exclusion of gastrointestinal perforation.

Clinical signs and symptoms might range from lethal arrhythmia to asymptomatic myocarditis. Ventricular and supraventricular ectopics, bundle branch block, and ST-T abnormalities are examples of electrocardiographic symptoms. Compared to traditional cardiac enzyme level testing, elevated blood levels of cardiac troponin-T more sensitively demonstrate myocyte damage in individuals with clinically suspected myocarditis^[^12,15^]^. The deranged cardiac enzymes showed signs of cardiac involvement in our patient, but on the seventh day after admission, they returned to normal. One infrequent sign of PPD poisoning that has been reported in the literature is myocardial involvement^[^16^]^.

PPD poisoning is a medical emergency with a significant death rate if it is not identified and treated quickly^[^16–18^]^. Although there is no precise remedy, the most crucial part of treatment is early detection and supportive measures, such as infusions of sodium bicarbonate, normal saline, and gastric lavage. The three main early problems are cardiac arrhythmias, myocarditis, and respiratory distress. Vigilant monitoring is necessary to avoid these conditions, which can lead to early fatalities. Asphyxia may necessitate intubation and ventilator assistance, and all dialysis modalities have been demonstrated to be beneficial in cases of renal failure^[^10^]^.

During an emergency, notably when patient history and good laboratory facilities are lacking, the characteristic angioedema of the face and neck, with difficulty in breathing and acute renal failure manifesting as chocolate brown-colored urine, could be suggestive of PPD poisoning^[^19–21^]^. Vigilance must be maintained regarding cardiac manifestations and other late complications of PPD poisoning for at least 1 month in all cases^[^9,22^]^.

## Conclusion

In conclusion, this case report underscores the urgency and severity of PPD poisoning, commonly associated with hair color use in the Asian subcontinent. Early detection and immediate supportive interventions are essential for patient survival. PPD ingestion can lead to angioedema, cardiac issues, hepatic dysfunction, and renal failure, emphasizing the importance of comprehensive monitoring. Healthcare providers should maintain vigilance in regions where PPD exposure is prevalent, and public awareness, along with product regulation measures, is necessary to mitigate the risks associated with PPD-containing products.

## Data Availability

Data available upon request from the authors.
